# Biliopancreatic Diversion is associated with greater increases in energy expenditure than Roux-en-Y Gastric Bypass

**DOI:** 10.1371/journal.pone.0194538

**Published:** 2018-04-04

**Authors:** Malin Werling, Lars Fändriks, Torsten Olbers, Tom Mala, Jon Kristinsson, Kaj Stenlöf, Ville Wallenius, Neil G. Docherty, Carel W. le Roux

**Affiliations:** 1 Department of Gastrosurgical Research and Education, Sahlgrenska academy, University of Gothenburg, Department of Surgery, Sahlgrenska University Hospital/Sahlgrenska, Gothenburg, Sweden; 2 Department of Morbid Obesity and Bariatric Surgery and Department of Gastrointestinal Surgery, Oslo University Hospital, Oslo, Norway; 3 Gothia Forum, Sahlgrenska University Hospital, Gothenburg, Sweden; 4 Diabetes Complications Research Centre, Conway Institute, University College Dublin, Dublin, Ireland; 5 Investigative Science, Imperial College London, London, United Kingdom; Western University of Health Sciences, UNITED STATES

## Abstract

**Objective:**

The greater weight loss achieved following Biliopancreatic Diversion with Duodenal Switch (BPDS) versus Roux-en-Y Gastric Bypass (RYGB) has been attributed to the malabsorptive effects of BPDS. Increased weight loss after BPDS could also be underpinned by larger increases in energy expenditure. Hypothetically, the more radical reconfiguration of the small intestine in BPDS could result in an accentuated increase in meal associated thermogenesis (MAT).

**Design:**

Female subjects (baseline mean age 40 years, mean BMI-55kg/m^2^) were assessed four years after randomization to BPDS (n = 6) or RYGB (n = 6). Energy expenditure (EE) and respiratory quotient (RQ) were measured by indirect calorimetry over 24 hours. A detailed protocol allowed for discrimination of basal metabolic rate (BMR), fasting EE and MAT as components of total energy expenditure (TEE) normalised for total and lean tissue by dual-energy x-ray absorptiometry.

**Results:**

Median weight loss at follow-up was 1.5-fold higher following BPDS relative to RYGB, resulting in respective median BMIs of 29.5 kg/m^2^ (21.7 to 36.7) after BPDS and 37.8 kg/m^2^ (34.1 to 45.7) after RYGB (p = 0.015). The BPDS group had a lower fat:lean ratio compared to the RYGB group (p = 0.009). Overall 24-hour TEE adjusted for total tissue was higher in the BPDS group, as were BMR, fasting EE and MAT (all p<0.05). Differences between RYGB and BPDS in BMR and TEE were nullified when normalised for lean mass. Postprandial RQ increased significantly but to a similar extent in both groups.

**Conclusion:**

Enhanced and prolonged MAT and lower fat:lean mass ratios after BPDS may explain relative increases in total energy expenditure as compared to RYGB.

## Introduction

Five-year follow up results from a clinical trial of 60 patients with body mass indices (BMI) between 50 and 60, randomised to undergo Biliopancreatic Diversion with Duodenal Switch (BPDS) or Roux-en-Y gastric bypass (RYGB) demonstrated a mean BMI reduction of 22.1 kg/m^2^ after BPDS and 13.1 kg/m^2^ after RYGB [1]. Lipid parameters and fasting glucose levels were also superior in BPDS operated subjects.

Weight loss after BPDS is often attributed to malabsorption [2–4] while weight loss after RYGB has been principally attributed to physiological adaptations impacting on metabolic control and satiety. Exaggerated post-prandial release of anorexigenic enteroendocrine hormones (e.g. L-cell glucagon like peptide-1) and L-cell hyperplasia have been implicated as mediators of reductions of food intake after RYGB. [5–8].

We recently added a new dimension to the suggested mechanisms of RYGB induced weight loss. We described progressive increases in post-prandial meal associated thermogenesis (MAT) and respiratory quotient (RQ) [9] that are not explainable by weight loss [10]. Complimentary studies in rodents support the inference that the source of the increased MAT is the alimentary limb of the Roux-en-Y reconstruction into which food rapidly transits [11, 12]. The rapid and unregulated exposure of the alimentary limb mucosa to nutrient in the absence of gastric pre-processing or biliopancreatic secretions may induce a hypertrophic mucosal response. This not only increases local enteroendocrine cell number but also increases post-prandial metabolic demand and MAT.

Thermogenic responses after BPDS are also predicted to be altered in a fundamentally similar way. Pre-clinical data in rats has demonstrated elevated dark-phase energy expenditure in rats undergoing this procedure relative to diet restricted weight matched sham operated rats [13]. Thus, MAT may be an important phenomenon after BPDS but could also be quantitatively distinct in nature to the changes found after RYGB, given that the intestinal bypass component of the procedure differs significantly from that found in RYGB. Typically, the alimentary limb after BPDS is approximately 33% longer than in RYGB (200cm versus 100-150cm) and is composed of jejunum and ileum rather than jejunum alone. Furthermore, the length of the biliopancreatic (BP) limb conveying unmixed biliopancreatic secretions is a standard length of 50cm in RYGB but significantly longer in BPDS. Finally the shorter common channel after BPDS may confer an increased adaptive pressure stimulus driving energy consuming hypertrophy [14].

Given the likelihood of increased adaptive pressure in the gut post-BPDS, we hypothesised that the procedure would be associated with a higher total energy expenditure (TEE) relative to RYGB. To test this, we studied 24-hour TEE and its constituent components- focussing in particular on MAT in in recipients of BPDS and RYGB drawn from the randomised trial described above [1, 15]

## Methods

### Ethics and participants

The study was approved by the Regional Ethical Review Board in Gothenburg, Sweden and performed in accordance with the Declaration of Helsinki. All study participants received information in person and in writing and provided written informed consent before participation in the study. The investigation was performed at the Department of Gastrosurgical Research & Education, Sahlgrenska University hospital, Gothenburg, Sweden.

Participants were recruited from a randomized clinical trial (Clinical Trials Identification Number-NCT00327912).conducted in collaboration between two academic clinical centres in Sweden and Norway. In the trial 60 patients with BMI 50 to 60 kg/m^2^ were randomized to either BPDS (n = 29) or RYGB (n = 31). Operations were performed during 2006 and 2007. Randomization was stratified according to study centre, sex, age and BMI. The present study included 12 female subjects from the Swedish cohort (six BPDS and six RYGB patients), examined at median four years after surgery; (Table 1). Participants that volunteered to complete the sub-study were not significantly different at baseline with regards to age, BMI or fat:lean ratios and all participants were examined at similar post-operative times. The sub-study populations did not differ in baseline or follow-up characteristics from the whole treatment group from which they were drawn and can thus be considered as representative. All participants were weight stable for at least three months before assessment and prescribed a multi vitamin and mineral supplementation tablet for at least two weeks prior to study participation.

**Table 1:** Time after surgery & weight loss.

	BPDS (n = 6)		RYGB (n = 6)		p-value
	Median	Range	Median	Range	
Time after surgery [years]	4.1	(3.6 to 4.5)	4.2	(3.5 to 7.3)	0.67
Age at assessment [years]	39	(32.1 to 47.6)	40.5	(24.6 to 46.5)	0.78
Body weight before surgery [kg]	147.5	(116.0 to 166.0)	160.3	(127–184)	0.33
Body weight after surgery [kg]	83.2	(55.0–93.7)	107	(99.7–130.9)	<0.01
Total weight loss [%]	48	(22.9 to 39.1)	31.7	(22.9 to 60.3)	0.03
BMI before surgery [kg/m^2^]	55.5	(47.6 to 58.9)	56.1	(47.2 to 59.4)	0.76
BMI after surgery [kg/m^2^]	29.5	(21.7 to 36.7)	37.8	(34.1 to 45.7)	0.02
Excess BMI loss [%]	86.1	(48.2 to 111.1)	55.8	(33.7 to 70.7)	0.03
Fat:Lean ratio before surgery	1.1	(0.9 to 1.2)	1.2	(0.7 to 1.2)	0.9
Fat:Lean ratio after surgery	0.55	(0.5 to 0.8)	0.9	(0.7 to 1.3)	0.08
Delta Lean mass (% of total tissue)	+13.2	(-5.5 to +16.7)	+2.6	(-6.9 to +9.6)	0.05
Delta Fat mass (% or total tissue)	-18.5	(-24.1 to -8.7)	-7.8	(-10.5 to 3.9)	0.02
Delta fat lean ratio after surgery	-0.51	(-0.7 to -0.07)	-0.2	(-0.4 to +0.3)	0.01

BPDS = Biliopancreatic diversion with duodenal switch. RYGB = Roux-en-Y gastric bypass. BMI = Body mass index.

### Surgery

Both the BPDS and RYGB were performed laparoscopically. The BPDS was created by a sleeve gastrectomy, stapled along a nasogastric tube of 30 to 32 Fr. The duodenum was divided shortly after the pylorus and the small intestine transected 300 cm from the ileocaecal valve. The distal part of the transected small intestine was anastomosed to the duodenal stump. The proximal part of the transected small intestine was anastomosed to the ileum 100 cm proximal to the ileocaecal valve. Thus, the total distance from the duodenal anastomosis to the ileocaecal valve was 300cm, of which 200cm constituted the alimentary limb and 100cm the common limb. The biliopancreatic limb consisted of the remaining long but unmeasured small intestine. In the RYGB procedure, the stomach was divided close to the oesophago-gastric junction creating a gastric pouch of about 25 mL and a gastric remnant. The jejunum was divided 50 cm aboral to the ligament of Treitz creating the biliopancreatic limb. The jejunum distal to the transection site was connected to the gastric pouch, creating the alimentary limb. The biliopancreatic limb was connected to the alimentary limb 150 cm distal to the gastric pouch. Mesenteric defects were not closed in either procedure.

### The metabolic chamber

Energy expenditure was measured by indirect calorimetry over 24 hours in a metabolic chamber, constructed as a small hotel room, 3x3x3 meters in dimension, including a bed, desk chair, desk, bookshelf, armchair, toilet, and washbasin. Fresh, dried and filtered air was circulated, while the temperature and humidity in the chamber was kept constant at 25°C and 40% relative humidity. Oxygen and carbon dioxide contents in exhausted air leaving the chamber were measured constantly enabling an assessment of energy expenditure (EE) and RQ. Patients entered the chamber before lunchtime on the first day of the study visit and exited after 25 hours. Thirty minutes at the beginning and 30 minutes at the end of the period were excluded from analysis to allow for acclimatization, settling after entrance and preparation before exiting. Thus, 24 hours were available for analysis.

### Study visit schedule (Fig 1)

The evening before study visit patients had a standardized dinner of mashed potatoes and meatballs at 19.00–20.00. The morning after, the patient arrived at the laboratory at 07.30 after an 11h fast. Weight and height were measured in light underwear. Dual Energy X-Ray Absorptiometry (DEXA) (LUNAR Radiation, Madison, WI, USA) was used to assess total, adipose and lean tissue as well as bone mass and density at study time. Baseline body composition was assessed using measurement of total body potassium based on assessment of gamma emission from endogenous intracellular natural potassium radioisotope (^40^K). This is considered as Gold Standard for assessment of lean mass but has been been variously reported to be in good agreement with DEXA based measurements using Bland-Altmann analysis[16]

The DEXA values were used to adjust recorded energy expenditure values for total or lean tissue during the metabolic chamber study.

**Fig 1:**
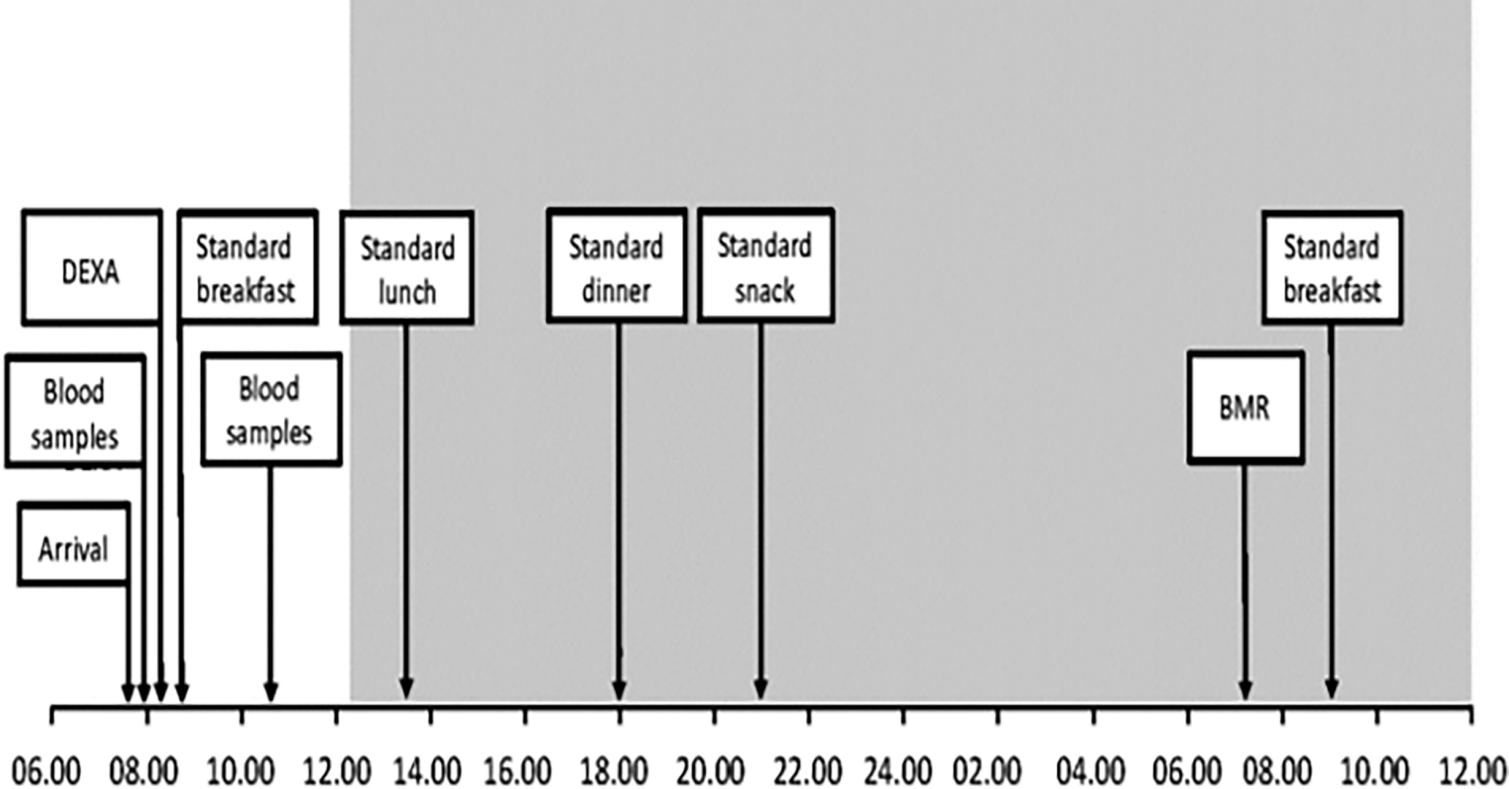
Study visit schedule.

Blood samples were collected in the fasting state for levels of free fatty acids, total cholesterols, high density lipoprotein (HDL), low density lipoprotein (LDL), iron, glycated haemoglobin (HbA1c), thyroid stimulating hormone (TSH), free thyroxin (fT4) and total triiodothyronine (T3), and follicle stimulating hormone (FSH) and creatinine.

During the first day of the study visit patients were served a 400-kcal standard breakfast (8% protein, 36% carbohydrate, 56% fat) at 09.00 before entering the chamber. While in the chamber, patients received four meals consisting of a 400-kcal fixed lunch (8% protein, 36% carbohydrate, 56% fat) at 13.30, a 600-kcal fixed dinner (meatballs and mashed potatoes; 600 kcal 13% protein, 41% carbohydrate, 46% fat) at 18.00 then one 120 kcal fixed evening snack at 21.00 and an *ad libitum* breakfast the following morning. Fixed caloric intake of 1520kcal during the period in the metabolic chamber was standard for all participants and the a ratio of intake (kcal) per unit of body mass (kg) was calculated.

Twenty-four-hour TEE was expressed as kcal/24 hours. When appropriate, EE was expressed as calories per minute over the full 24h study period adjusted for total or lean tissue (kg) as assessed by DEXA scan.

Basal metabolic rate (BMR) was assessed on the second day of the study visit. Patients were woken at 07.00 and allowed to go to the bathroom then instructed to get back into bed. From 07.30 to 08.30 patients were lying supine and awake in bed looking at the ceiling without detectable movements of the arms or legs. The EE recorded during the 30 minutes between 07.45 and 08.15 was used for BMR analyses.

Fasting EE and changes in EE after food intake were analysed either side of dinner at 18.00. Participants were instructed to consume the meal within 30 minutes of it being served at 18.00. As our objective was to test the hypothesis that gut thermogenesis may differ between the procedures, the 30-minute period from 18.00 to 18.30 during which food was being consumed was excluded from post-prandial EE analysis in order to avoid inclusion of physical activity associated with meal consumption. Fasting EE was analysed between 17.00 and 18.00 immediately prior to the meal and values obtained were considered as the gross composite of BMR and non-exercise associated thermogenesis. Changes in EE versus fasting were assessed between 18.30 and 19.30 and from 19.30 to 20.30. MAT was defined as a positive change in EE above the fasting EE baseline occurring after food intake. RQ was calculated alongside EE from the ratio between total CO_2_ production and O_2_ consumed.

Physical activity was assessed during the full duration of the stay in the metabolic chamber. Assessment was based on the percentage of the total chamber study time during which the study participant broke infrared light beams strategically placed in the chamber.

### Statistical analyses

Group data (including mean per minute EE data for individual subjects) was summarised using non-parametric description with median and range recorded unless otherwise stated. The Mann Whitney two-tailed exact test was used for comparison between groups. The conventional p<0.05 was used as the statistical rejection criterion. A power analysis made on data from a previous study on bariatric patients using the same protocol and measuring equipment determined that for an alpha of 0.05 and beta of 0.2 we should use a group replication of 6 to compare TEE.

## Results

### Body composition (Table 1)

The study was conducted during the weight-stable phase after both procedures and median (range) reduction in BMI after BPDS versus baseline was 48%, representing a BMI reduction from 55.5 kg/m^2^ (47.6 to 58.9) to 29.5 kg/m^2^ (21.7 to 36.7). After RYGB the BMI reduction was 31.7% with BMI falling from 56.1 kg/m^2^ (47.2 to 59.4) to 37.8 kg/m^2^ (34.1 to 45.7). Median BMI reduction following BPDS was thus 51% greater than after RYGB (p = 0.015). The median fat:lean ratios were 37.7% lower in the BPDS group relative to the RYGB group; 0.55 (0.49 to 0.79) versus 0.91 (0.74 to 1.34) respectively (p = 0.008). Calculation of intra-individual changes in fat mass and lean mass as proportions of total tissue between pre-operative assessment and follow-up emphasise that weight loss and changes in fat:lean ration derive mainly from loss of adipose tissue, this being quantitatively greater following BPDS.

The mean reductions in BMI and mean F:L ratio values for recipients of each intervention included in the sub-study did not significantly different at 5-year follow-up from the data recorded for the wider parent RCT RCT population (S1 and S2 Tables).

Differences in absolute body weight at 5 years between the BPDS and RYGB groups resulted in a differential ratio of fixed calorie intake (kcal) to body weight (kg) during the metabolic chamber study. After BPDS there was a higher ratio of intake to body mass over fixed meal intake; BPDS-16.3 kcal/kg(range 16.2–27.6) versus RYGB 14.2 (range 11.6–15.2) p = 0.002.

### Clinical factors (S3 Table)

Clinical data on diagnoses and medication were available for participants within one year of the study visit (S 3 Table).

### Fasting metabolic profile (Table 2)

Participants receiving BPDS had lower total cholesterol, LDL-cholesterol, HbA1c and HDL-cholesterol relative to recipients of RYGB. There was no evidence of differences in thyroid function or ovarian cycle and thus both factors could be excluded as confounders in the assessment of energy expenditure in subsequent analyses.

**Table 2:** Biochemical variables.

	BPDS (n = 6)		RYGB (n = 6)		p-value
	Median	Range	Median	Range	
**Fasting samples [units] (reference values)**					
Free fatty acid [mmol/L] (0.45–2.6)	0.8	(0.6 to 1.3)	0.8	(0.1 to 1.2)	0.99
Cholesterol [mmol/L] (2.9–6.1)	3.2	(2.7 to 4.5)	4.4	(3.9 to 5.1)	0.03
HDL [mmol/L] (1–2.7)	1.2	(0.96 to 1.4)	1.5	(1.3 to 1.7)	0.02
LDL [mmol/L] (1.2–4.3)	1.7	(1.4 to 2.6)	2.7	(1.9 to 3.3)	0.03
Iron [μmol/L] (9–34)	10	(8 to 26)	6.5	(3 to 27)	0.28
Hb [g/L] (117–153)	124	(111 to 134)	115.5	(74 to 147)	0.39
HbA1c [mmol/mol] (27–42)	26.5	(24 to 34)	36	(30 to 38)	0.04
Free T4 [pmol/L] (12–22)	16	(13 to 17)	15	(12 to 16)	0.15
TSH [mIU/L] (0.3–4.2)	1.9	(1.4 to 2.8)	1.8	(1.3 to 5.8)	0.73
FSH [IU/L]	7.2	(2.9 to 29)	4.4	(2.3 to 5.5)	0.09

Values are median (Range). Mann Whitney two-tailed exact test were used.

BPDS = Biliopancreatic diversion with duodenal switch. RYGB = Roux-en-Y gastric bypass.

HDL = High-density lipoprotein. LDL = Low density lipoprotein. Hb = hemoglobin. HbA1c = Glycated hemoglobin. T4 = ….TSH = Thyroid-stimulating hormone. FSH = Follicle stimulating hormone

### Physical activity, respiratory quotient and energy expenditure (Fig 2 and Table 3)

Physical activity during the 24-hour study period was comparable between the BPDS and RYGB patients; 5.4% (3.1 to 8.9) and 6.1% (3.8 to 9.6) (p = 0.48)

**Fig 2:**
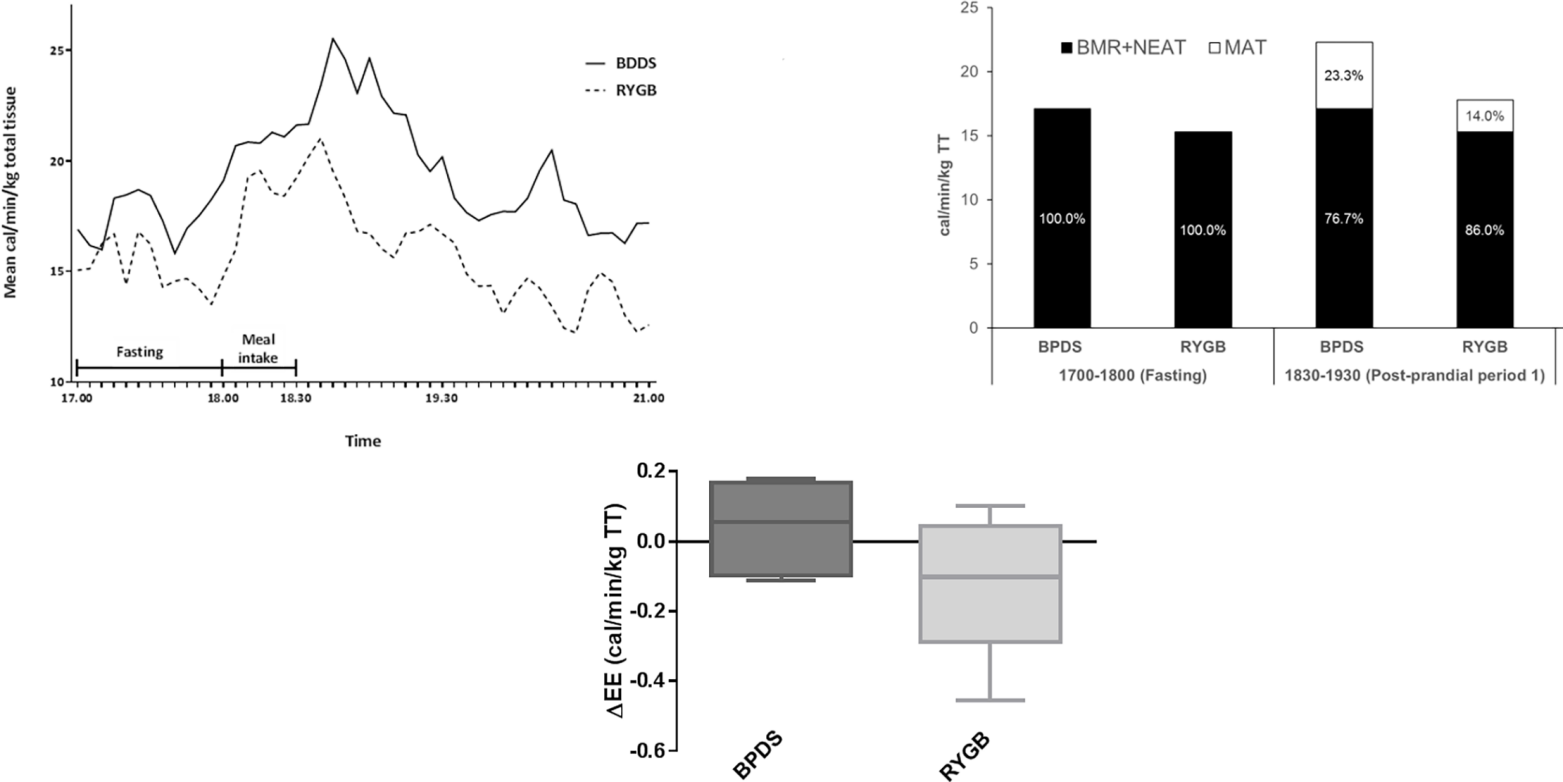
Fasting and post-prandial energy expenditure. A) Mean energy expenditure adjusted for total tissue in fasting and after standard meal four years after Biliopancreatic diversion with duodenal switch (BPDS, n = 6) or Roux-en-Y gastric bypass (RYGB, n = 6). B) Summary energy expenditure data for BPDS and RYGB groups during fasting and at 2 post-prandial periods. Mean energy expenditure at each period is presented in cal/min/kg total tissue (TT). The relative percentage proportions (%) of TT deriving from non-exercise associated thermogenesis (BMR+NEAT) and meal associated thermogenesis (MAT) are illustrated. C) Change in EE versus fasting during second post-prandial hour. See Table 3 for group summary data and inter-group statistical comparison.

**Table 3:** Energy expenditure, respiratory quotient and diet induced thermogenesis.

	BPDS (n = 6)		RYGB (n = 6)		p-value
	Median	Range	Median	Range	
**Total 24 hour data**					
EE [kcal/min]	1.3	(1.1 to 1.5)	1.5	(1.3 to 1.9)	0.30
EE [cal/min/kg total tissue]	16.9	(14.1 to 23.7)	14.1	(10.8 to 15.2)	0.01
EE [cal/min/kg lean tissue]	27.2	(24.5 to 37.2)	26.1	(24.2 to 30.5)	0.78
RQ	0.8	(0.7 to 0.9)	0.8	(0.7 to 0.9)	0.78
**BMR, awake in lying position, between 07.45 and 08.15 h**					
EE [kcal/min]	1.1	(0.9 to 1.3)	1.2	(0.8 to 1.6)	0.68
EE [cal/min/kg total tissue]	14.3	(11.0 to 17.6)	11.5	(7.2 to 13.5)	0.04
EE [cal/min/kg lean tissue]	22.5	(19.3 to 27.6)	21.2	(16.7 to 26.9)	0.68
RQ	0.8	(0.7 to 0.9)	0.8	(0,7 to 1.0)	0.39
**Fasting EE during 1 hour before standard meal; between 17:00 and 18:00 h**					
EE [kcal/min]	1.4	(1.0 to 1.5)	1.7	(1.4 to 2.2)	0.04
EE [cal/min/kg total tissue]	17.1	(15.0 to 22.9)	15.3	(13.7 to 16.8)	0.09
RQ	0.8	(0.7 to 0.8)	0.8	(0.7 to 0.8)	0.78
**EE during first hour after standard meal; between 18.30 and 19.30 h**					
EE [kcal/min]	1.7	(1.4 to 2.1)	1.9	(1.7 to 2.5)	0.39
EE [cal/min/kg total tissue]	22.3	(19.1 to 25.5)	17.8	(15.3 to 19.4)	<0.01
MAT [kcal/min]	0,4	(0,2 to 0,7)	0,3	(0,2 to 0,4)	0.48
MAT [cal/min/kg total tissue]	5,2	(2,1 to 7,2)	2,3	(1,6 to 3,5)	0.04
RQ	0.9	(0.8 to 0.9)	0.8	(0.8 to 0.9)	0.81
**EE during second hour after standard meal; between 19.30 and 20.30 h**					
EE [kcal/min]	1.4	(1.0 to 1.6)	1.6	(1.1 to 2.2)	0.58
EE [cal/min/kg total tissue]	17.21	(13.9 to 25.1)	14.2	(9.7 to 17.0)	0.04
Change versus Fasting [kcal/min]	0.06	(-0.11 to 0.18)	-0.10	(-0.46 to 0.10)	0.18
Change versus Fasting [cal/min/kg total tissue]	0.85	(-1.26 to 2.23)	-0.85	(-3.95 to 0.98)	0.18
RQ	0.9	(0.8 to 0.9)	0.8	(0.8 to 0.9)	0.48

Mann Whitney two-tailed exact test were used.

BPDS = Biliopancreatic diversion with duodenal switch. RYGB = Roux-en-Y gastric bypass.

EE = energy expenditure. RQ = respiratory quotient. MAT = meal associated thermogenesis (EE minus Pre-meal fasting EE = change versus fasting. The term MAT is not used for the second post-prandial hour due to the presence of negative values versus fasting EE in both groups. Standard meal; 600 kcal ingested during 30 minutes.

There were no between group differences regarding RQ analysed during the full 24-hour period (p = 0.81), during BMR measurement (p = 039) or in the fasting (p = 0.72) or post-prandial state (p = 0.48). However an elevation in RQ from 0.78 (0.67 to 0.83) to 0.84 (0.81 to 0.92) for the BPDS group (p = 0.03) and from 0.75 (0.72 to 0.81) to 0.83 (0.77 to 0.92) for the RYGB group (p = 0.03) was evident between the fasting and fed state demonstrating a shift from fat to carbohydrate oxidation.

When adjusted for total tissue, the BPDS group had higher median 24-hour TEE as compared to the RYGB patients; 16.9 cal/min/kg (14.1 to 23.7) and 14.1 cal/min/kg (10.8 to 15.2), respectively (p = 0.015). The BPDS group also had a 24% higher total tissue normalised median of mean BMR; 14.3 cal/min/kg (11.0 to 17.5) and 11.5 cal/min/kg (7.2 to 13.5), respectively (p = 0.041). When adjusting for lean tissue there was no between group difference for 24-hour TEE (p = 0.78) or BMR (p = 0.67).

Adjusted for total tissue, the fasting median of mean EE was 17.09 cal/min/kg total tissue (15.02 to 22.86] for BPDS and 15.34 cal/min/kg total tissue (13.66 to 16.8] for RYGB (p = 0.093). During the first post-prandial hour from 18.30 to 19.30 total tissue normalised median of mean EE and MAT were 26% (p = 0.004) and 225% (p = 0.04) higher in the BPDS group relative to the RYGB group respectively. During the second post-prandial hour from 19.30 to 20.30 total tissue normalised median of mean EE did not differ significantly versus fasting levels for either group. Unadjusted EE in the fasting state was higher in the RYGB group as compared to the BPDS group (p = 0.04).

## Discussion

In the present study we predicted that the thermogenic response to eating would be enhanced more after BPDS as compared to RYGB, resulting in relative increases in total tissue adjusted 24-hour energy expenditure. We hypothesised that this difference would arise as a logical consequence of the more extensive intestinal bypass in BPDS.

Different bariatric procedures vary in efficacy with regard to magnitude and durability of weight loss [17]. We previously demonstrated that RYGB is associated with an increase in MAT relative to vertical banded gastroplasty [10] and that post-operative increases in MAT can be tracked longitudinally in individual patients [9]. These observations could support the contention that the intestinal bypass component of RYGB augments weight-loss in part through an enhancement of MAT. However, the causual relationship between the differences in TEE observed and long-term weight loss remains to be further explored.

In line with predictions, a relative increase in MAT was observed in the first post-prandial hour after BPDS. Relative to RYGB, The common channel is much shorter after BPDS and the BP limb is much longer. This difference could be anticipated to confer a differential degree of adaptive pressure in the common channel after BPDS. The limited length of the BPDS common channel coupled to the likelihood that as a consequence of the long length of the BP-limb, mixing of biliopancreatic secretions with food in the common channel is not well synchronised, this may drive energy consumption post-prandially and long-term mucosal hypertrophic adaptations.

Studies in rodents show that the gut undergoes a hypertrophic response after RYGB and BPDS [7, 8, 14]. Increases in proliferation of the alimentary limb mucosa have also been evidenced in humans after RYGB [18, 19]. Perspectives on how relatively modest increases in alimentary limb length in BPDS relative to RYGB might disproportionately translate into increases in MAT can be drawn from studies in rodents. Increases in metabolic demand accompany hypertrophy, and in the alimentary limb this adaptive drive is more energetically challenged due to the combined effects of more rapid nutrient transfer and the absence of biliopancreatic sections. The absence of sodium containing biliopancreatic secretions (for sodium coupled glucose uptake) and reductions in sodium coupled glucose transporter 1 (SGLT-1) expression have been implicated in reduced alimentary limb capture of luminal glucose [20, 21]. A switch to glucose extraction from the circulation and its glycolytic disposal in the alimentary limb has been demonstrated in rats, a phenomenon which may have dual anti-hyperglycaemic and thermogenic effects that exceed what could be anticipated from consideration of purely the length of bowel involved [11]. We suspect that bile exclusion from the alimentary limb also limits the potential for the small intestine to switch to fatty acid uptake driven beta-oxidation as a means of supporting the hypertrophic response, further emphasising the importance of glucose extraction from the circulation.

Importantly however even if increasing the alimentary limb does create a proportional increase in MAT, it would seem unlikely that this would be the dominant force guiding relative increases in weight loss. Weight loss at follow-up did not differ between standard and distal RYGB in which BP-limb length was maintained at equivalence [22].

Unexpectedly we noted a relative increase in total body weight adjusted BMR in BPDS versus RYGB. Fat:lean mass ratios were lower after BPDS. Thus, relative increases in the proportion of skeletal muscle in recipients of BPDS may contribute to augmented total weight normalised energy expenditure relative to RYGB. As differences in EE between BPDS and RYGB were nullified when adjusted for lean mass rather than total mass, skeletal muscle thermogenic activity is probably not intrinsically different between the procedures. Differences in brown-fat thermogenesis as a contributory factor to our observations cannot be excluded. However, the markedly increased postprandial RQ speaks against a dominant role for brown adipose tissue as being a dominant feature of MAT [23].

In this study TEE increased more after BPDS as compared to RYGB likely through a combination of a more marked increases in MAT and BMR. Limitations with respect to the conclusions that can be drawn include the very low levels of physical activity, some differences between groups with regard to the post-operative time (years) at which the study took place and the fact that the study only included females. Moreover, our study being small and cross-sectional is limited in its ability to interrogate whether subtle differences in fat: lean ratios and percentage weight loss can be discretely assigned as causes or consequences of changes in energy expenditure. Differences in proportional fixed meal challenge (kcal per kg body weight) can also not be entirely ruled out as an explanatory component of differences observed in meal associated thermogenesis. Whether the apparent increased prevalence of co-morbidities after BPDS observed in our sub-study (S3 Table) has any bearing on increased energy expenditure after BPDS would require examination in a larger scale study in which an inflammatory disease comorbidity composite could be entered as an independent variable into multivariable regression analysis.

All things considered however, the study demonstrates differences in weight normalised energy expenditure between RYGB and BPDS and suggests that these differences may support increased weight loss and weight loss maintenance after BPDS. With specific reference to meal associated thermogenesis, the data bring forward the hypothesis that frequent consumption of small meals may through a summative effect, make a significant contribution to 24 hour energy expenditure and hence be operative as a driver of weight loss after bariatric surgery.

## Supporting information

S1 Table 5-year follow-up body weight and BMI data. (DOCX)

S2 Table 5 year Follow-up fat:Lean ratios. (DOCX)

S3 Table Co-morbidities and medications. (DOCX)

S1 File Anonymised minimal dataset. (XLSX)
