# Supplementary material for: Biliopancreatic Diversion is associated with greater increases in energy expenditure than Roux-en-Y Gastric Bypass
Source: PLoS One. 2018 Apr 4;13(4):e0194538. doi: 10.1371/journal.pone.0194538 (PMC5884508; doi:10.1371/journal.pone.0194538)
Supplement: S1 Table — (DOCX) [file pone.0194538.s001.docx]

**Table S1: 5-year follow-up body weight and BMI data**

| **RYGB** | **PRE-OP** | | | **5-YEAR** | | | **DELTAS** | |
| --- | --- | --- | --- | --- | --- | --- | --- | --- |
|  | height (m) | weight (kg) | BMI (kg/m2) | height (m) | weight (kg) | BMI (kg/m2) | DELTA WEIGHT | DELTA BMI |
| ANON | 1.8 | 184.0 | 59.4 | 1.8 | 130.9 | 42.7 | -53.1 | -16.7 |
| ANON | 1.6 | 140.3 | 56.2 | 1.6 | 106.0 | 42.5 | -34.3 | -13.7 |
| ANON | 1.7 | 164.5 | 56.9 | 1.7 | 108.6 | 37.6 | -55.9 | -19.3 |
| ANON | 1.6 | 127.0 | 47.2 | 1.6 | 106.0 | 39.4 | -21.0 | -7.8 |
| ANON | 1.7 | 156.0 | 56.0 | 1.6 | 99.7 | 37.1 | -56.3 | -18.9 |
| ANON | 1.8 | 182.0 | 56.2 | 1.8 | 108.0 | 33.0 | -74.0 | -23.2 |
|  | | | | | | | **MEAN SUB-STUDY** | **-16.6** |
|  |  |  |  |  |  |  | **MEAN RCT** | **13.6 (95% CI 11.0 to 16.1)** |
|  | | | | | | | | |
| **BPDS** | **PRE-OP** | | | **5-YEAR** | | | **DELTAS** | |
|  | height (m) | weight (kg) | BMI (kg/m2) | height (m) | weight (kg) | BMI (kg/m2) | DELTA WEIGHT | DELTA BMI |
| ANON | 1.6 | 140.0 | 54.7 | 1.6 | 55.0 | 21.4 | -85.0 | -33.2 |
| ANON | 1.6 | 155.0 | 58.3 | 1.6 | 8.01 | 30.4 | -74.0 | -27.8 |
| ANON | 1.7 | 164.5 | 54.9 | 1.7 | 85.3 | 28.1 | -79.2 | -26.8 |
| ANON | 1.56 | 116.00 | 47.67 | 1.56 | 93 | 38.2149901 | -23.00 | -9.45 |
| ANON | 1.72 | 166.00 | 56.11 | 1.7 | 93.7 | 32.4221453 | -72.30 | -23.69 |
| ANON | 1.51 | 134.50 | 58.99 | 1.51 | 68.7 | 30.1302574 | -65.80 | -28.86 |
|  | | | | | | | **MEAN SUB-STUDY** | **-25.0** |
|  |  |  |  |  |  |  | **MEAN RCT** | **22.7 (95% CI 19.5 to 24.7)** |
