# Supplementary material for: Biliopancreatic Diversion is associated with greater increases in energy expenditure than Roux-en-Y Gastric Bypass
Source: PLoS One. 2018 Apr 4;13(4):e0194538. doi: 10.1371/journal.pone.0194538 (PMC5884508; doi:10.1371/journal.pone.0194538)
Supplement: S2 Table — (DOCX) [file pone.0194538.s002.docx]

**S2 Table: 5 year Follow-up fat:lean ratios**

| **WERLING ET AL 2017 5 YEAR DATA** | | | | | | | |
| --- | --- | --- | --- | --- | --- | --- | --- |
| **OTHER SWEDISH NCT00327912 5 YEAR DATA** | | | | | | | |
| **RYGB** |  | | | **BPDS** |  |  |  |
|  | Lean Mass (Kg) | Fat Mass (Kg) | F:L RATIO |  | Lean Mass (Kg) | Fat Mass (Kg) | F:L RATIO |
| ANON | 65.5 | 62.3 | 0.95 | ANON | 35.4 | 18.4 | 0.52 |
| ANON | 48.7 | 55.8 | 1.15 | ANON | 50.3 | 29.1 | 0.58 |
| ANON | 53.5 | 52.2 | 0.98 | ANON | 55.6 | 27.2 | 0.49 |
| ANON | 56.5 | 46.5 | 0.82 | ANON | 48.3 | 42.9 | 0.89 |
| ANON | 52.5 | 45.3 | 0.86 | ANON | 58.9 | 33.2 | 0.56 |
| ANON | 57.3 | 48.7 | 0.85 | ANON | 38.4 | 28.1 | 0.73 |
| ANON | 68 | 43 | 0.63 | ANON | 48.4 | 41.3 | 0.85 |
| ANON | 58.6 | 44 | 0.75 | ANON | 50.2 | 26.2 | 0.52 |
| ANON | 55.3 | 39.6 | 0.72 | ANON | 72.5 | 34 | 0.47 |
| ANON | 58.3 | 53.6 | 0.92 | ANON | 63.6 | 35 | 0.55 |
| ANON | 61.9 | 58.6 | 0.95 | ANON | 75.9 | 27.1 | 0.36 |
| ANON | 85.5 | 79.2 | 0.93 | ANON | 55.4 | 62.5 | 1.13 |
| ANON | 75.9 | 71.3 | 0.94 | ANON | 67.6 | 27.8 | 0.41 |
|  | **SUB-STUDY RYGB F:L MEAN (SD)** | | **0.93 (0.12)** | ANON | 48.5 | 22.4 | 0.46 |
|  | **ALL SWEDISH RYGB F:L MEAN (SD)** | | **0.88 (0.13)** |  | **SUB-STUDY BPDS F:L MEAN (SD)** | | **0.63 (0.15)** |
|  | **T-TEST SUB STUDY V OTHERS** | | **p=0.17** |  | **ALL SWEDISH BPDS F:L MEAN (SD)** | | **0.61 (0.22)** |
|  |  | | |  | **T-TEST SUB STUDY V OTHERS** | | **p=0.76** |
