# Supplementary material for: Biliopancreatic Diversion is associated with greater increases in energy expenditure than Roux-en-Y Gastric Bypass
Source: PLoS One. 2018 Apr 4;13(4):e0194538. doi: 10.1371/journal.pone.0194538 (PMC5884508; doi:10.1371/journal.pone.0194538)
Supplement: S3 Table — (DOCX) [file pone.0194538.s003.docx]

**S3 Table: Co-morbidities and medications**

| **Group** | **Subject** | **Co-morbidities** | **Medications** |
| --- | --- | --- | --- |
| RYGB | 1 | None | Vitamin B12 |
|  | 2 | None | Vitamin B12 |
|  | 3 | None | Vitamin B12 |
|  | 4 | Infertility | Vitamin B12, multivitamin, Calcium, IVF hormonal therapy |
|  | 5 | None | Vitamin B12, Calcium |
|  | 6 | Nausea | Omeprazol |
| BPDS | 1 | Fibromyalgia | Folate, calcium, Vitamin B12, vitamin D + Paracetamol daily |
|  | 2 | None | Vitamin D, Calcium, Vitamin B12, Iron, omega3 substition |
|  | 3 | Gastro-oesophageal reflux, gout, joint disease, gallstone | Multivitamin, Vitamin B12, Calcium, Iron |
|  | 4 | Joint disease, depression | Vitamin B12, calcium, NSAID, Omeprazol |
|  | 5 | None | Multivitamin |
|  | 6 | Gastro-oesophageal reflux, joint disease | Multivitamin, Iron, Calcium, Folate Omeprazol |
